# Chloride, glutathiones, and insect-derived elicitors introduced into the xylem trigger electrical signaling

**DOI:** 10.1093/plphys/kiad584

**Published:** 2023-10-31

**Authors:** Yong-Qiang Gao, Hugo Morin, Laurence Marcourt, Tsu-Hao Yang, Jean-Luc Wolfender, Edward E Farmer

**Affiliations:** Department of Plant Molecular Biology, University of Lausanne, Lausanne 1015, Switzerland; Institute of Pharmaceutical Sciences of Western Switzerland, University of Geneva, CMU, Geneva 1206, Switzerland; School of Pharmaceutical Science, University of Geneva, CMU, Geneva 1206, Switzerland; Institute of Pharmaceutical Sciences of Western Switzerland, University of Geneva, CMU, Geneva 1206, Switzerland; School of Pharmaceutical Science, University of Geneva, CMU, Geneva 1206, Switzerland; Department of Plant Molecular Biology, University of Lausanne, Lausanne 1015, Switzerland; Institute of Pharmaceutical Sciences of Western Switzerland, University of Geneva, CMU, Geneva 1206, Switzerland; School of Pharmaceutical Science, University of Geneva, CMU, Geneva 1206, Switzerland; Department of Plant Molecular Biology, University of Lausanne, Lausanne 1015, Switzerland

## Abstract

Ricca assays allow the direct introduction of compounds extracted from plants or the organisms that attack them into the leaf vasculature. Using chromatographic fractionation of Arabidopsis (*Arabidopsis thaliana*) leaf extracts, we found glutamate was the most active low mass elicitor of membrane depolarization. However, other known elicitors of membrane depolarization are generated in the wound response. These include unstable aglycones generated by glucosinolate (GSL) breakdown. None of the aglycone-derived GSL-breakdown products, including nitriles and isothiocyanates, that we tested using Ricca assays triggered electrical activity. Instead, we found that glutathione and the GSL-derived compound sulforaphane glutathione triggered membrane depolarizations. These findings identify a potential link between GSL breakdown and glutathione in the generation of membrane depolarizing signals. Noting that the chromatographic fractionation of plant extracts can dilute or exchange ions, we found that Cl^−^ caused glutamate receptor-like3.3-dependent membrane depolarizations. In summary, we show that, in addition to glutamate, glutathione derivatives as well as chloride ions will need to be considered as potential elicitors of wound-response membrane potential change. Finally, by introducing aphid (*Brevicoryne brassicae*) extracts or the flagellin-derived peptide flg22 into the leaf vasculature we extend the use of Ricca assays for the exploration of insect/plant and bacteria/plant interactions.

## Teaser

Using a century-old method it is possible to introduce diverse molecules of non-plant origin into the vasculature in intact leaves.

## Introduction

A method pioneered by Ricca (1916) allows the introduction of molecules into the living vasculature of plants. With this technique, compounds of interest can be transferred from a damaged leaf into the veins of distant, intact leaves. The first step in the procedure involves burning or scalding petioles in order to kill all cells surrounding xylem vessels. The damaged part of the petiole can then be cut into any solution from which water and solutes are carried through the xylem transpiration stream in a basipetal direction toward the stem. The fluid stream then enters undamaged distal leaves and moves acropetally toward their tips. Based on the use of this method, Ricca (1916) postulated that “hormones” derived from wounded parts of *Mimosa* species moved through the xylem to trigger distal leaf movements. The subsequent association of leaf movements and electrical signals in *Mimosa* (e.g. Umrath 1928) eventually led to the term “Ricca's factors” being generalized to molecules that activate electrical signaling in wounded plants (Sambeek and Pickard 1976). Over the years, a number of unsuccessful attempts were made to identify components of leaf extracts that triggered membrane depolarizations in plants (e.g. Cheeseman and Pickard 1977; Sibaoka 1997). Recently, Ricca assays were employed in renewed attempts to identify Ricca's factors in slow wave potential (SWP) signaling in Arabidopsis (*Arabidopsis thaliana*, Gao et al. 2023).

The SWP electrical signal in an undamaged Arabidopsis leaf distal to a wounded leaf has a complex architecture. A rapid membrane depolarization phase is followed by a long-duration irregular repolarization phase typically lasting approximately 2 min and occasionally displaying repetitive depolarization spikes (Mousavi et al. 2013). The ability to propagate SWPs from a wounded leaf to a distal leaf depends on clade 3 glutamate receptor-like (GLR) proteins, and in particular on GLR3.3 and GLR3.6 (Mousavi et al. 2013). The use of the Ricca assay combined with genetic approaches revealed that two β-thioglucoside glucohydrolases (TGG1 and TGG2) were the principal Ricca's factors in adult-phase Arabidopsis (Gao et al. 2023). Mutants lacking TGG1 and TGG2, or plants lacking aliphatic glucosinolates (GSLs) which are substrates for these enzymes, failed to produce wild-type (WT)-like SWPs. A mechanism was proposed in which TGG-catalyzed GSL breakdown generated short-lived aglycone (thiohydroximate-*O*-sulfonate) elicitors of membrane depolarization. However, in *tgg1 tgg2* double mutants, further activities remained. These other membrane depolarizing factors likely include glutamate (Shao et al. 2020; Gao et al. 2023) a molecule strongly implicated in wound-response calcium signaling (Toyota et al. 2018; Bellandi et al. 2022; Grenzi et al. 2023). In principle, stable membrane depolarizing elicitors such as glutamate could be extracted, fractionated, assayed, and purified from plants. However, such standard approaches are not without their limitations. For example, some active factors might be short-lived and therefore difficult to purify—as is the case for unstable aglucones derived from GSL breakdown (Mocniak et al. 2020). During extraction, other active compounds might react with cell components that they would not encounter in undamaged tissues. Additionally, ions with biological activities may be diluted-out during fractionation of plant extracts. Finally, many plant–insect and plant pathogen interactions could liberate or generate compounds capable of affecting plant membrane potentials. We took each of these possibilities into account in a search for further elicitors of membrane depolarization in *A. thaliana*. In addition to employing electrophysiology assay-driven fractionation of Arabidopsis leaf extracts we investigated nonplant-derived substances for their ability to trigger membrane potential changes when introduced into the xylem.

## Results

### Introduction of molecules into the leaf vasculature

For Ricca assays, a pipette tip fitted onto a 5 mL syringe (Supplemental Figure S1A) was used to drip boiling water (approximately 0.5 mL) onto the leaf 8 petiole of a 5-wk-old plant. A toothpick channeled excess water into the soil (Supplemental Figure S1B). While room temperature water did not elicit electrical activity in leaf 8 or leaf 13, the scalding procedure triggered repetitive depolarizations in leaf 8 (Supplemental Figure S1C). This procedure caused a low level of induction of *JASMONATE-ZIM-DOMAIN 10* (*JAZ10*) transcripts (which indicate jasmonate-response gene expression) in distal leaf 13. Levels of *JAZ10* transcripts induced by scalding in leaf 13 were about 4-fold higher than those of untreated plants (Supplemental Figure S1D). Fluorescein was used to trace molecular movement from leaf 8 to distal leaf 13. Fluorescein transport from leaf 8 to leaf 13 occurred to a similar extent and at a similar velocity when the healthy leaf 8 petiole was severed, or when the equivalent region of a scalded petiole was cut (Supplemental Fig. S2, Supplemental Movies 1 and 2). This indicated that scalding and cutting through dead tissue did not disrupt fluid movement compared to cutting through living petiole tissue.

Using this procedure, we investigated how quickly biologically active substances needed to be introduced into the scalded petiole in order for the assay to function. For these experiments, fresh leaf extract (FLE) was prepared. Using the experimental set-up shown in Supplemental Figure S3A, the scalded region of the leaf 8 petiole was either cut in FLE immediately or cut into a buffer then FLE was added into the buffer at various times after cutting. For over 25 min after first cutting into a buffer, FLE introduction into the buffer elicited electrical signals in the “local” scalded leaf. However, in distal leaf 13 we noted a progressive decay in electrical signal duration in the first 25 min after cutting the scalded region. In this distal leaf, signal amplitudes were almost invariably in the region of −75 to −30 mV or else they were near zero (Supplemental Figure S3B). In the 1,500 s after cutting the scalded region the apparent velocity of the electrical signal through the distal leaf 13 remained in the region of 5 to 14 (average 10) cm/min (Supplemental Figure S3C). In conclusion, FLE can be introduced into the scalded petiole and electrical signals excited in this same leaf 8 petiole for at least 30 min after initial incubation in a buffer. By contrast, we found that the cut petiole must be treated with an elicitor solution within no more than 3 min of cutting in order to trigger long-duration depolarizations in the distal leaf 13. In all subsequent experiments, the scalded region of leaf 8 was cut immediately into test solutions.

### Fractionation of leaf extracts prior to Ricca assays

**Figure 1.**
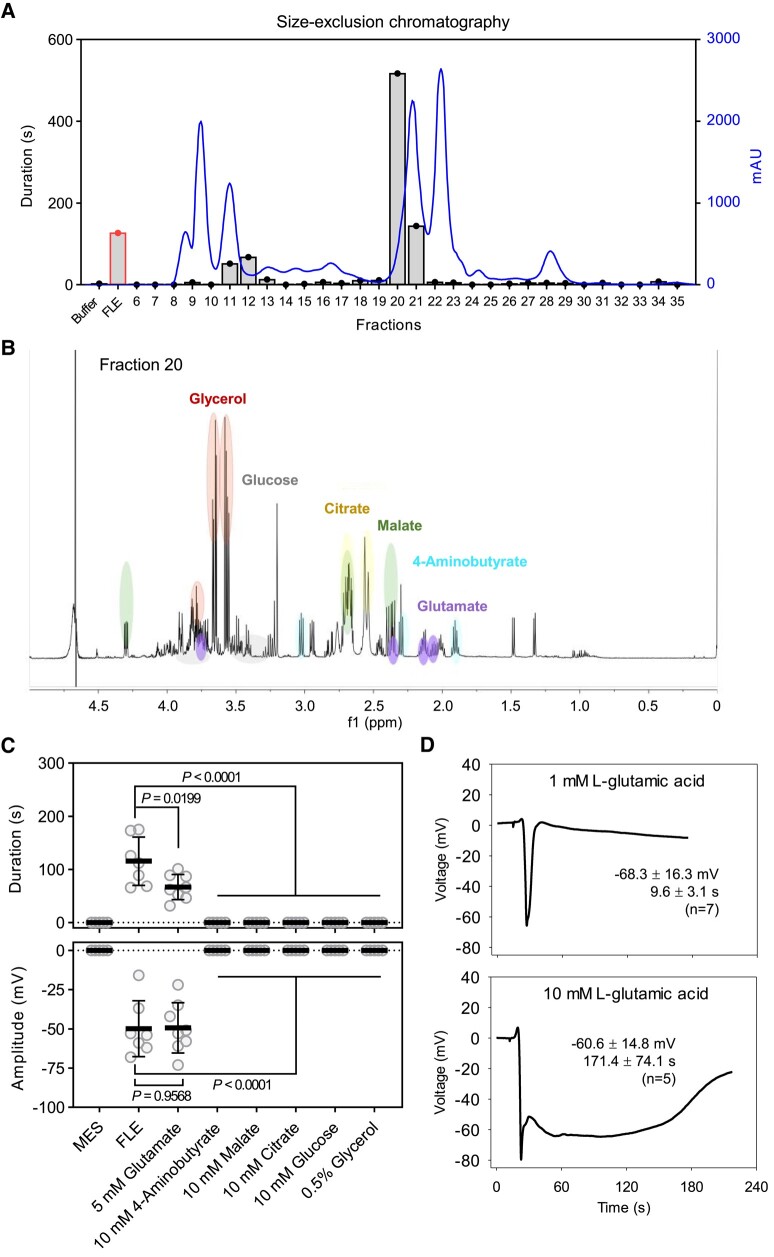
Fractionation of SWP-inducing compounds in FLE. **A)** SEC profile of FLE and activity of fractions. The activity was tested by using Ricca assays and shown as the duration of electrical signals (*n* = 1 to 3). **B)**^1^H NMR spectrum of the active fraction 20 obtained after SEC. The characteristic signals of glutamate are highlighted, the other ^1^H NMR signals were attributed based on a comparison with the Chenomx spectral reference libraries. **C)** The activity of candidates (*n* = 17, means ± SD, unpaired two-tailed Student's *t*-test). All compounds were dissolved in 50 mM MES, pH 6.0 with Tris. **D)** Typical recordings of glutamate (L-glutamic acid)-induced electrical signals. Data in each graph indicate amplitude (mV), duration (s), and the number of replicates (means ± SD). Chemicals were applied from leaf 8 and SWPs were recorded on leaf 13 with the surface electrodes. The significance threshold was *P* < 0.05.

Genetic studies of electrical signaling in Arabidopsis leaves distal to wounds have shown that the β-thioglucoside glucohydrolases, TGG1 and TGG2, are the most active elicitors of membrane depolarization in leaves distal to wounds (Gao et al. 2023). To identify further components of leaf extracts which could trigger membrane depolarization, FLE was fractionated by size-exclusion chromatography (SEC). Fractions eluted from the SEC column were then tested for their activity in the Ricca assay. Two activity peaks were identified. Fractions 11 and 12 in Fig. 1A corresponded to previously characterized TGG1 and TGG2 proteins (Gao et al. 2023). Fractions 20 and 21 were enriched in low molecular weight compounds. Fraction 20 triggered membrane depolarizations of exceptionally long duration (over 8 min) which was longer than the duration of the SWP triggered by wounding (Mousavi et al. 2013). Nuclear magnetic resonance (NMR) analysis of this fraction identified six principal components: glycerol, glucose, citrate, malate, glutamate, and 4-aminobutyrate (Fig. 1B). Despite several overlaps with other signals, the annotated protons of glutamic acid were recovered in fraction 20 (Supplemental Fig. S4). In pure form, each of the six compounds detected in fraction 20 was assayed for its ability to trigger membrane depolarization in the Ricca assay. Only glutamate was active in these tests (Fig. 1C). The activity of glutamate in the assay was concentration-dependent. At 1 mM concentration glutamate provoked a rapid, spike-like depolarization. However, when its concentration was increased to 10 mM, glutamate triggered a spike depolarization followed by a prolonged depolarization phase reminiscent of a slow wave potential (Fig. 1D). The activity of the other 19 canonical amino acids were compared to that of glutamate in the Ricca assay. In some assays, cysteine (Cys) provoked short, spike-like depolarizations (Supplemental Fig. S5). Previously, L-Cys was found to be more active than Glu in triggering cytosolic Ca^2+^ transients in Arabidopsis roots (Alfieri et al. 2020). Together, these findings drew our attention to other S-containing molecules and among which were GSL breakdown products.

### Effects of sulfur-containing compounds on membrane potentials

**Figure 2.**
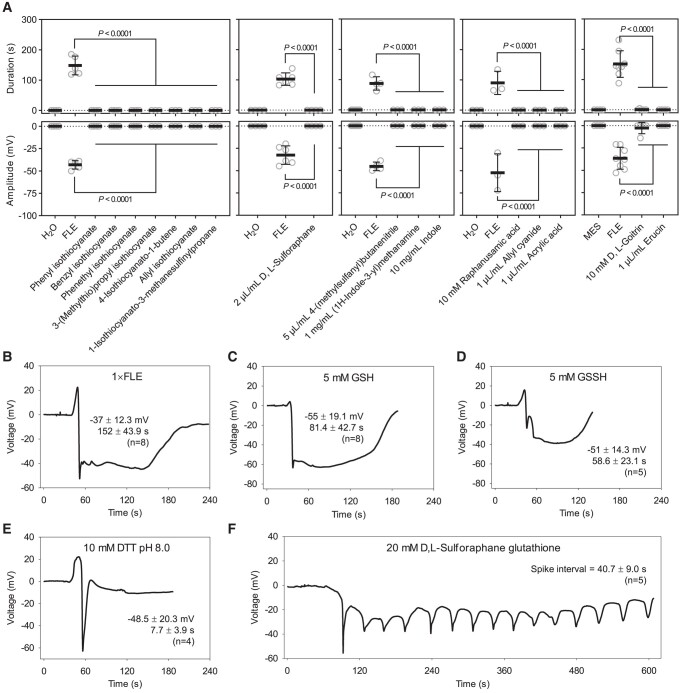
Effect of stable sulfur-containing molecules on membrane depolarization. **A)** GSL breakdown products and other compounds tested (*n* = 3 to 8, means ± SD, unpaired two-tailed Student's *t*-test). Except when indicated, compounds were used at 0.1 μL/mL. **B–F)**. Typical recording of 1 × FLE **B)**, 5 mM GSH **C)**, 5 mM GSSH **D)**, 10 mM DTT **E)**, and **D)**, L-sulforaphane glutathione (F)-induced electrical signals. FLE = fresh leaf extract; GSH = reduced glutathione; GSSH = oxidized glutathione. Data in each graph indicate amplitude (mV), duration (s), and the number of replicates (means ± SD). Compounds were dissolved in H_2_O or 50 mM MES, pH 6.0 with Tris, except DTT was dissolved in 50 mM Tris, pH 8.0 with HCl. Chemicals were applied from leaf 8 and SWPs were recorded on leaf 13 with the surface electrodes. The significance threshold was *P* < 0.05.

The breakdown of aliphatic GSLs by TGG1 and TGG2 is essential for slow wave potential signaling in Arabidopsis (Gao et al. 2023). GSL breakdown can be envisaged as a two-step process. Firstly, hydrolysis of the glucose moiety produces unstable aglucones which have short half-lives (Mocniak et al. 2020). These compounds can then decay producing more stable derivatives including isothiocyanates and nitriles (Wittstock and Burow 2010). In the current model, aglucones produced by the breakdown of aliphatic GSLs directly or indirectly trigger wound-response membrane depolarization (Gao et al. 2023). We tested a variety of other GSL breakdown products for their activities in the Ricca assay. However, none of the 16 compounds tested, which ranged from isothiocyanates to nitriles, etc. was active at concentrations shown in Fig. 2A. The electrophilic isothiocyanate sulforaphane was inactive in the assay (Fig. 2A). However, isothiocyanates can be coupled to the glutamate-containing molecule glutathione (GSH; Yagishita et al. 2019). This led us to investigate the activity of GSH and related compounds.

Using the Ricca assay, reduced GSH and oxidized GSSH were, like FLE (Fig. 2B), active in triggering membrane depolarizations in Arabidopsis (Fig. 2, C and D). When we tested the laboratory reducing agent dithiothreitol (DTT) we found that it could trigger spike-like depolarization of membranes in distal leaf 13 (Fig. 2E). However, the architecture of the DTT-triggered signal differed to the architectures of the GSH- and GSSH-induced signals which displayed more prolonged repolarization phases. Knowing that GSL-derived aglycones act directly or indirectly in SWP signaling (Gao et al. 2023), and having detected membrane depolarization activities with GSH, we decided to test the potential biological activity of sulforaphane glutathione. This conjugate is one of several metabolites of the aliphatic GSL glucoraphanin. Sulforaphane glutathione at a concentration of 20 mM elicited repetitive spike depolarizations (Fig. 2F).

Slow wave potential signals activate the synthesis of defense-inducing jasmonates such as jasmonoyl-isoleucine (Mousavi et al. 2013). When introduced into the vasculature, do GSH and its derivates cause activation of the jasmonate pathway? To test this GSH or GSSH (each at 5 mM) or sulforaphane glutathione (20 mM) were introduced into the leaf 8 petiole. One hour later leaf 13 was sampled and *JAZ10* transcript levels were measured in that leaf. Each GSH derivative induced *JAZ10* transcript accumulation (Supplemental Fig. S6). Next, we considered GSH mutants. Since loss-of-function GSH mutants are embryo-lethal (e.g. Cairns et al. 2006), we tested SWP generation in the *phytoalexin-deficient 2-1* (*pad2-1*) mutant which contains only 22% of WT GSH levels. Under our pest-free culture conditions, *pad2-1* grows similarly to the WT (Parisy et al. 2007). Leaf 8 of *pad2-1* was wounded and SWPs were measured in distal leaf 13. The SWPs were slightly affected in the mutant plants (Supplemental Fig. S7).

### Effect of ions on membrane depolarization

**Figure 3.**
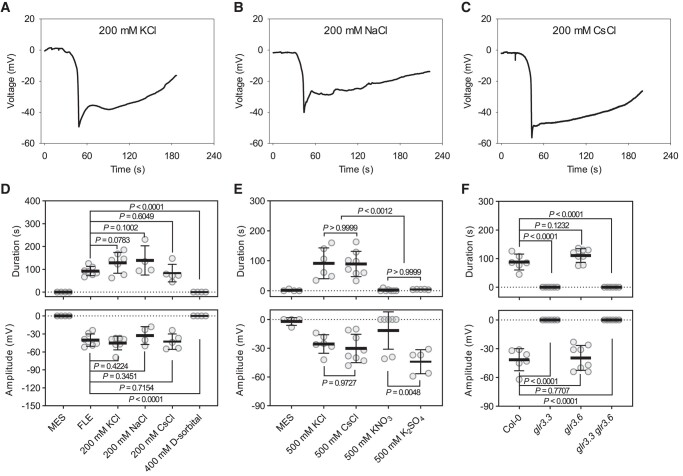
Long-duration chloride-induced electrical signals. **A)** Typical recording of 200 mM KCl-induced electrical signal. **B)** Typical recording of 200 mM NaCl-induced electrical signal. **C)** Typical recording of 200 mM CsCl-induced electrical signal. **D)** 200 mM chloride salt-induced electrical signals (*n* = 4 to 7, means ± SD, unpaired two-tailed Student's *t*-test). **E)** 500 mM Cl^−^/NO_3_^−^/SO_4_^2−^ salt-induced electrical signals (*n* = 4 to 8, means ± SD, one way ANOVA followed by Tukey's test for multiple comparisons). **F)** 500 mM CsCl-induced electrical signals in *glr* mutants (*n* = 6 to 8, means ± SD, unpaired two-tailed Student's *t*-test). All salts were dissolved in 50 mM MES, pH 6.0 with Tris. Chemicals were applied from leaf 8 and slow wave potentials (SWPs) were recorded on leaf 13 with the surface electrodes. The significance threshold was *P* < 0.05.

Many ions which are abundant in plant cells could, in theory, be diluted or exchanged during chromatographic fractionation. K^+^ has signaling functions and is of central importance in plant electrophysiology (Britto and Kronzucker 2008). When we tested 200 mM KCl in the Ricca assay we found that it triggered membrane depolarizations which resembled the SWPs but lacked the apparent hyperpolarization which precedes the depolarization phase of the SWP (Fig. 3A). Expecting membrane depolarization to be due to K^+^, we tested NaCl and CsCl at the same concentration as a control. In both cases NaCl and CsCl provoked membrane depolarizations (Fig. 3, B and C). To rule out osmotic effects, sorbitol was tested in the assay. This compound did not induce membrane depolarization (Fig. 3D). However, two potassium salts, K_2_SO_4_, and to a lesser extent KNO_3_, stimulated the production of short, spike-like depolarizations (Fig. 3E). The finding that the Cl^−^ anion could trigger membrane depolarization was unexpected and prompted us to investigate whether mutations in two of the principal *GLR* genes involved in the SWP signaling (GLR3.3 and GLR3.6) affected the plants ability to respond to this ion. In these experiments *glr3.3*, *glr3.6*, and *glr3.3 glr3.6* mutants were treated with 500 mM CsCl, in each case using Ricca assays to feed the salt into leaf 8. Interestingly, CsCl introduced into leaf 8 failed to trigger electrical activity in leaf 13 of the *glr3.3* single mutant or the *glr3.3 glr3.6* double mutant, whereas electrical activity in the *glr3.6* single mutant resembled that elicited in the WT (Fig. 3F). Having found that the Cl^−^ anion can trigger membrane depolarizations we investigated SWP production in several CHLORIDE CHANNEL and ALUMINUM-ACTIVATED MALATE TRANSPORTER mutants (Supplemental Table S1). None of the plants we tested strongly affected SWP production.

### Membrane depolarizing activities from a herbivorous insect and a bacterial pathogen

**Figure 4.**
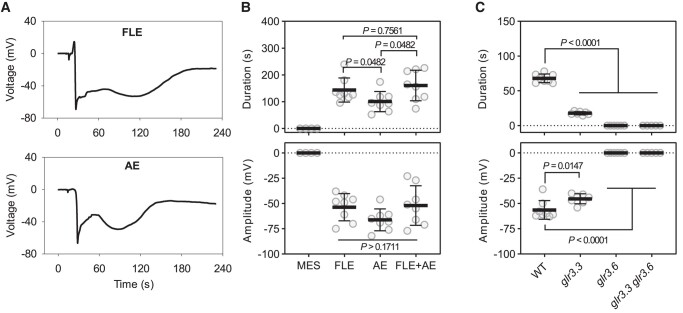
The activity of aphid extract. **A)** Typical electrical signal recordings after stimulation with FLE (upper panels) and AE (lower panels). **B)** AE-induced electrical signals (*n* = 4 to 8, means ± SD, one way ANOVA followed by Tukey's test for multiple comparisons). FLE and AE was diluted to 0.5× (final) in 50 mM MES, pH 6.0 with Tris to test for activity. **C)** AE-induced electrical signals in *glr* mutants (*n* = 5 to 8, means ± SD, unpaired two-tailed Student's *t*-test). Aphid extract was applied from leaf 8 and SWPs were recorded on leaf 13 with the surface electrodes. The significance threshold was *P* < 0.05.

Elicitors derived from pathogens have long been known to affect membrane potentials (e.g. Pelissier et al. 1986) and the same is true of some insect-derived factors (e.g. Maffei et al. 2004; Camoni et al. 2018). Among effector-secreting insects (Tanaka and Heil 2021) are vein-feeding hemipterans. To test whether Ricca assays could be used to study insect-derived factors we produced and tested an extract from the phloem-feeding aphid *Brevicoryne brassicae*. This extract triggered long-duration depolarizations which are compared to those elicited by FLE in Fig. 4, A and B. Having found biological activity in aphid extract (AE) we then investigated whether this was GLR-dependent. Remarkably, *glr3.3* and *glr3.6* mutants differentially affected membrane depolarizations triggered by AE. Specifically, *glr3.3* strongly reduced the duration of the depolarization and had a less pronounced effect on signal amplitude (Fig. 4C). By contrast, the *glr3.6* single mutant abolished all responses to the extract. Electrical signal production in response to the AE was also abolished in the *glr3.3 glr3.6* double mutant.

**Figure 5.**
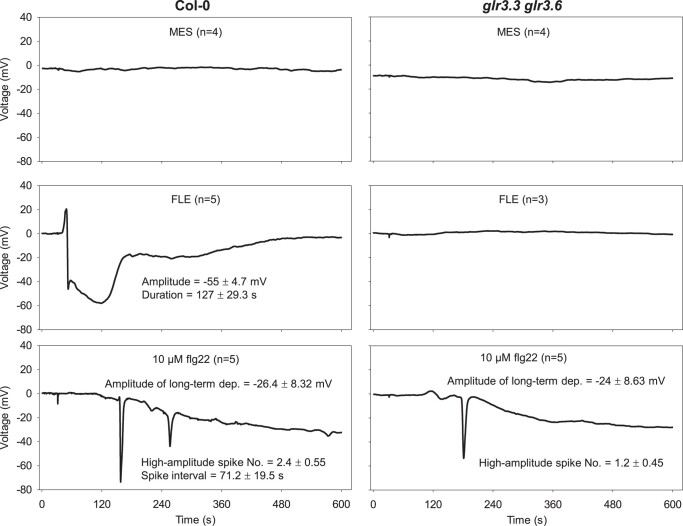
The activity of a bacterial PAMP. Electrical signals induced by flg22. 1× FLE or 10 μM flg22 were applied to wild-type Col-0 (left) and *glr3.3 glr3.6* (right). *n* = number of replicates; No. = mean number of spikes; dep. = depolarization. flg22 was diluted with 50 mM MES buffered to pH 6.0 with Tris. Chemicals were applied from leaf 8 and SWPs were recorded on leaf 13 with the surface electrodes.

Finally, we tested the bacterial flagellin-derived pathogen-associated molecular pattern (PAMP) flg22. flg22 triggers immune responses in plants (DeFalco and Zipfel 2021). When introduced into the leaf 8 xylem of the WT, flg22 elicited spike-like depolarizations in distal leaf 13 very different from those induced by FLE (Fig. 5). In addition, flg22 induced a gradual reduction in membrane potential. This gradual weakening in membrane potential was not affected substantially in the *glr3.3 glr3.6* double mutant background. Additionally, the double mutant did not affect the major depolarization detected but attenuated secondary spiking activity elicited by flg22.

## Discussion

Loss-of-function *tgg* mutants have confirmed that TGGs are the major Ricca's factors acting to generate membrane depolarization elicitors in leaves distal to wounds in Arabidopsis (Gao et al. 2023). However, *tgg* mutants did not eliminate all electrical activity in leaves distal to wounds and different compounds are likely to contribute to membrane depolarization in the wounded leaf itself. Glutamate is the best candidate for such a molecule, but are there further compounds that need consideration? In their search for plant-derived elicitors of leaf movement or membrane depolarization, some researchers have used boiled or solvent-extracted plant extracts (e.g. Fitting 1936; Sambeek and Pickard 1976). We avoided these treatments and analyzed FLEs. At the outset we took two potential weaknesses of assay-based fractionation into consideration. Firstly, fractionation and/or exchange into buffers might dilute biologically relevant ions. Secondly, mixing of reactive cellular components (even in the absence of enzyme activity) could, in theory, remove some active molecular species and generate others. Our findings reveal that, both Cl^−^ anions and the cellular protectant glutathione (GSH/GSSH) will need to be investigated for their potential contributions to wound-induced membrane depolarizations.

### Highly active low mass fractions contain glutamate

Previous work identified high mass (TGG protein) elicitors of membrane depolarization in Arabidopsis leaves distal to wounds (Gao et al. 2023). In this work, we fractionated leaf extracts using the SEC and focused on lower mass elicitors. When eluted fractions containing low molecular weight compounds were assayed for their ability to provoke membrane potential changes the most active fraction contained a mixture of organic acids including glutamate (Fig. 1B). Glutamate proved to be the only compound in the fraction which triggered membrane depolarization, and this was strongly concentration-dependent (Fig. 1, C and D). These results are consistent with early work on the leaf movement-provoking activity of glutamate in *Mimosa pudica* (Fitting 1936; Schildknecht et al. 1978), with more recent work on the ability of glutamate to induce increases in cytosolic Ca^2+^ levels in Arabidopsis (Toyota et al. 2018; Bellandi et al. 2022; Gao et al. 2023; Grenzi et al. 2023) as well as with the effects of exogenous glutamate on membrane potentials in this plant (Shao et al. 2020; Gao et al. 2023). In the Ricca assay, 1 mM glutamate triggered spike depolarizations in leaf 13 whereas 5 or 10 mM glutamate triggered long-duration depolarizations resembling slow wave potentials (Fig. 1D). The concentration-dependence of glutamate activity is particularly interesting and, at high concentrations, glutamate may be excitotoxic to plant cells (Gao and Farmer 2023). Consistent with the studies of Bellandi et al. (2022) and Grenzi et al. (2023), we speculate that glutamate might act as a membrane depolarization elicitor via gating of GLR3.3. However, other amino acids can act as GLR3.3 agonists to depolarize membranes in roots (Qi et al. 2006). To test their effects on leaf membrane potentials we, therefore, fed each canonical amino acid into the vasculature.

### S-containing molecules can act as modulators of membrane potential

In addition to the effects of glutamate, we noted that cysteine had weak activity in the Ricca assay where it triggered spike-like depolarizations of short-duration (Supplemental Fig. S5). Cysteine, like glutamate, is a ligand for GLR3.3 (Alfieri et al. 2020). We tested whether other S-containing molecules could influence membrane potentials when introduced into the vasculature. GSH is an abundant endogenous elicitor which can enhance defense gene expression in bean cell suspension cultures (Wingate et al. 1988). Interestingly, both GSH- and GSSH-induced depolarizations resembled SWPs in that they triggered rapid depolarizations followed by longer repolarization phases (Fig. 2, C and D). DTT also induced membrane depolarization (Fig. 2E). To test whether this might be a general property of S-containing molecules, we chose to study the compounds most closely related to the GSL-derived aglucones identified as TGG-produced depolarization-inducing factors (Gao et al. 2023). GSL breakdown produces a diversity of byproducts (Halkier and Gershenzon 2006; Wittstock and Burow 2010). Although we only tested a range of these molecules and did not investigate, for example, the potential activities of epithionitriles, none of the 16 GSL-related compounds tested, including nitriles and electrophilic isothiocyanates, triggered membrane potential changes (Fig. 2A). The compounds tested included benzyl isothiocyanate which suppresses proton pump phosphorylation and inhibits stomatal opening (Aihara et al. 2023). In our experiments, the failure to find effects of a variety of commercially available GSL breakdown products suggests that only sulfur (S) atoms in specific molecular contexts (e.g. linked to glutamate-containing molecules) may affect membrane potential when introduced into the vasculature.

Sulforaphane glutathione is a GSL-derived molecule with known activities in defense induction (Andersson et al. 2015). At high concentration (20 mM) this compound triggered repetitive spike depolarizations (Fig. 2F) which were reminiscent of electrical signals occasionally observed in wounded plants (Mousavi et al. 2013). In our study, pulsed spikes were sometimes observed with GSH or GSSH treatments, and they differed from the single spike depolarization typical of DTT treatment. We speculate that the repetitive spike depolarizations triggered by sulforaphane glutathione may arise through a different mechanism than that responsible for the protracted SWP depolarization phase.

We note that when concentrations as low as 20 mM glucoraphanin (the parent GSL from which sulforaphane glutathione is derived) were supplied to plants in the presence of TGG1 they triggered SWP-like membrane depolarizations (Gao et al. 2023). The three-orders-of-magnitude difference in activity between glucoraphanin and sulforaphane glutathione suggests that the aglucone-derived regulator(s) of membrane depolarization in leaves distal to wounds is/are likely to have a far higher activity than sulforaphane glutathione. The possibility that GSL-derived aglucones react directly with membrane proteins has been evoked (Gao et al. 2023). The xylem of plants in the Brassicales contains GSH (Mendoza-Cózatl et al. 2008) and it will be worthwhile investigating the possibility that aglucones derived from GSL breakdown in the xylem react with GSH or GSSH to form biologically active conjugates. Wound-response membrane depolarization causes activation of the jasmonate pathway (Mousavi et al. 2013) and GSH has been implicated previously in the control of basal jasmonate pathway activity (Han et al. 2013). When introduced into the vasculature, GSH, GSSH, and sulforaphane glutathione all stimulated *JAZ10* expression (Supplemental Fig. S6). However, the *pad2-1* mutant which has lower-than-WT GSH levels did not reduce SWP durations or amplitudes. Under our culture conditions, *pad2-1* produces enough GSH for healthy growth and seed production. We, therefore, do not rule out roles of GSH in electrical signaling which might be revealed in mutants with further reduced GSH levels. Additionally, we do not rule out possible effects on cellular redox potentials being linked to depolarization.

### Chloride as a regulator

Plant cells contain high millimolar levels of K^+^ and Cl^−^ ions (Clarkson and Hanson 1980). The fractionation and assay of plant extracts can dilute these ions. Therefore, prior to analyses of leaf extracts we investigated the effects of high concentrations of salts on membrane potentials in leaves. As expected, KCl (200 mM) triggered membrane depolarization (Fig. 3A) and we assumed initially that this was due to K^+^, an abundant cation important for the regulation of membrane potential (Britto and Kronzucker 2008; Shabala 2017) and which plays roles in long distance signaling (e.g. Wegner and De Boer 1997; Cuin et al. 2018). Unexpectedly, we found instead that Cl^−^ triggered membrane depolarization (Fig. 3, B and C). Consistent with this, and even at very high concentrations (500 mM), KNO_3_ and K_2_SO_4_ failed to trigger the long-duration depolarizations (Fig. 3E) that we observed with 200 mM KCl, NaCl or CsCl treatments. This was not due to ionic strength (*I*) since the KNO_3_ and K_2_SO_4_ solutions used had higher *I* (0.5 and 1.5, respectively) than the KCl or NaCl solutions used (*I* = 0.2). Remarkably, Cl^−^ induced depolarization was GLR3.3-dependent (Fig. 3F). H^+^-ATPase inactivation in response to wounding has been proposed (Julien et al. 1991). How Cl^−^ fed into the vasculature causes membrane depolarization is unknown and the few anion channels we tested failed to affect SWP production. Finding that Cl^−^, an anion with important regulatory properties (Valdivieso and Santa-Coloma 2019), can trigger membrane depolarization highlights the difficulty of using genetic approaches for quantitating the relative contributions of key ions, amino acids such as glutamate, and also GSH, all of which are essential cell components.

## Conclusions

The main advantage of using Ricca assays to probe biological activities is that substances enter a leaf that has not been damaged. Furthermore, these substances travel along a natural transport route, the transpiration stream. This delivery method has advantages since the vascular bundle may block the entry of certain substances infiltrated into leaves. Ricca assays, therefore, have great potential in studying vascular physiology and pathophysiology. A potential disadvantage is that substances fed into the vasculature will be diluted in the transpiration stream, as they move to distal leaves. This means that assessing the effective concentration of a substance at its site of action in vivo will be difficult. A further consideration is the site of action of molecules or ions introduced in the vasculature. Whether they act on xylem contact cells or, as has been evoked recently (Gao et al. 2023), may travel into the phloem region prior to activating GLR-dependent electrical signaling is unknown.

In addition to studying the effects of plant-derived factors, we found that insect- or pathogen-derived molecules can be introduced into the leaf vasculature using Ricca assays. To demonstrate this, we used extracts from phloem-feeding aphids as well as the bacterial PAMP flg22. Results from these experiments were of particular interest and showed that the effects of nonplant-derived substances on membrane potentials can be studied in the WT and mutant plants. Moreover, the procedures we used could now be combined with fractionation and employed, for example, to identify effectors from insects that feed on the vasculature. Equally interesting would be to employ Ricca assays to investigate the biology of effectors produced by vascular pathogens some of which cause serious yield losses (e.g. Burbank and Roper 2021). We, therefore, broaden the use of the term “Ricca's factors” to membrane potential-changing substances that are naturally released into the xylem from plants themselves or from the organisms that attack them. Given their versatility, Ricca assays could also find further uses in the introduction of hormones into veins; this may be of use to study vascular differentiation.

Finally, it might even be possible to use Ricca assays to introduce small quantities of molecules which can be replicated in cells surrounding xylem vessels. This could include, for example, the genomes of viral pathogens, or certain other nucleic acids.

## Materials and methods

### Plants and chemicals

Wild type Arabidopsis (*A. thaliana*) and all T-DNA insertion mutants were in Columbia (Col) genetic background and were obtained from the Nottingham Arabidopsis Stock Centre (http://arabidopsis.info/). The anion channel mutants we used are listed in Supplemental Table S2 and the *glr* mutants used were: *glr3.3* (SALK_099757), *glr3.6* (SALK_ 091801), and *glr3.3 glr3.6* (SALK_099757, SALK_ 091801; Mousavi et al. 2013). The γ-glutamylcysteine synthetase (γ-ECS, GSH1) mutant *pad2-1* was from Parisy et al. (2007). Seeds were planted on soil in 7 cm diameter pots and grown with 70% relative humidity, 10 h light (100 to 120 μEm^−2^ s^−1^) at 22 °C and 14 h dark at 18 °C. Chemicals were from Sigma-Aldrich (Buchs, Switzerland), except when specially indicated. Erucin (Adipogen SA, Liestal, Switzerland) and D,L-sulforaphane glutathione (Santa Cruz Biotechnology, Europe). Synthetic flg22 oligopeptide was from Peptide Specialty Laboratories GmbH (https://www.peptid.de/).

### The preparation of FLE and AE

The expanded rosette leaves from 5- to 6-wk-old WT *Arabidopsis* plants were collected for FLE preparation. Leaves were ground to powder in liquid nitrogen with a mortar and centrifuged at 12,000 × *g* at 10 °C for 10 min. The supernatant after centrifugation was collected and is referred to as FLE. Aphids (*B. brassicae*) feeding on cabbage (*Brassica oleracea*) were collected and ground to powder in liquid nitrogen with a mortar. Aphid powder (50 mg) was extracted in a 2 mL Eppendorf tube with 800 μL MES buffer (50 mM MES, pH 6.0 with Tris, prechilled to 4 °C) by gently inverting the tube ∼20 times, then centrifuged at 12,000 × *g* at 4 °C for 10 min, the supernatant was collected and is referred to as AE.

### Size-exclusion chromatography

A Superdex 200 increase 10/300 column (Cytiva, Glattbrugg, Switzerland) was washed with two column volumes of milli-Q water. The column was pre-equilibrated with 2.5-bed volumes of running buffer (2 mM MOPS, 200 mM KNO_3_, pH 7.5 with NaOH). Fresh leaf extract (0.5 mL) was flowed through a 0.22 μm sterile syringe filter (Cobetter Filtration Equipment Co., Ltd, Hangzhou, China), then injected into the column. Fractionation was performed at ∼8 °C at 1 mL/min flow rate with 1.5-bed volumes of running buffer. The eluent was collected at 1 mL/fraction and used for Ricca assays.

### Nuclear magnetic resonance spectroscopy

The most active fraction (fraction 20) from SEC was subjected to NMR analysis for the determination of its composition. This fraction was evaporated to dryness using an RVC 2-18 CDplus vacuum concentrator (CHRIST, Osterode am Harz, Germany) at room temperature. The dried fraction was dissolved in 500 µL of D_2_O and 100 µL of phosphate buffer solution (203 mM Na_2_HPO_4_, 44 mM NaH_2_PO_4_ (pH 7.4), 1 mM TSP, 3 mM NaN_3_) in order to minimize chemical shifts variation due to pH shifts. NMR spectra were recorded on a Bruker Avance Neo 600 MHz NMR spectrometer equipped with a QCI 5mmCryoprobe and a SampleJet automated sample changer (Bruker BioSpin, Rheinstetten, Germany). Chemical shifts are reported in parts per million (*δ*) using the residual D_2_O signal as an internal standard. Coupling constants (*J*) are given in Hz. A demo version of Chenomx NMR suite 9.0 (Chenomx Inc., Edmonton, Canada) was used to attribute chemical shifts to most of the ^1^H NMR signals detected. Among these signals, typical ^1^H NMR signals characteristic of glutamate were recorded. To further confirmed the attribution of the glutamate signals, a commercial standard of glutamic acid (2.5 mg) (Sigma-Aldrich, St. Louis, MO, USA) was analyzed under the same conditions. The corresponding ^1^H NMR signals for glutamic acid (phosphate buffer solution + D_2_O, 600 MHz) were: *δ* 2.07 (1H, m, H-3″), 2.14 (1H, m, H-3′), 2.36 (2H, m, H2-4), 3.76 (1H, dd, *J* = 7.3, 4.7 Hz, H-2). These signals matched the one attributed to glutamate in the active fraction 20 which were also partially overlapped with signals of other constituents in this fraction. For this, the individual ^1^H NMR pattern of each glutamate signal was compared.

### Ricca assays

Ricca assays were performed according to Gao et al. (2023). Five- to six-wk-old *Arabidopsis* plants were used for all experiments. Leaf 8 petiole was scalded by rapidly pipetting boiling water onto the petiole as shown in Supplemental Figure S1B. Around 5 mm of petiole was scalded and excess water on the petiole was removed with tissue paper. To ensure that the petiole is thoroughly scalded, the scalding procedure can be repeated. Then the scalded plants were rested under light at 22 °C for at least 3 h. To apply FLE or chemicals of interest, a vessel containing 60 to 70 μL of test solution was placed under the scalded petiole. The scalded section was then cut with a surgical scissors so that the solution entered the severed vasculature.

### Fluorescence imaging

An SMZ18 stereomicroscope (Nikon Instruments Europe BV, Amsterdam, Netherlands) equipped with an ORCA-Flash4.0 (C11440) camera (Hamamatsu, Solothurn, Switzerland) and an F36-525 eGFP filter set (excitation 472 ± 30 nm, emission 520 ± 35 nm; AHF analysentechnik AG, Tübingen, Germany) was used for fluorescence imaging. A SOLA SE II light engine (Nikon Instruments Europe BV, Amsterdam, Netherlands) was used as a light source. The excitation energy and lookup tables (LUTs; 60 to 300) were the same in each experiment. Intact or scalded leaf 8 petioles of 5- to 6-wk-old plants were cut in sodium fluorescein solution (NaFluo, 1 mg mL^−1^; Sigma-Aldrich, Buchs, Switzerland). Systemic propagation of fluorescence after cutting was acquired using NIS-Elements software (Nikon) with a resolution of 512 × 512 pixels in each frame (1 frame s^−1^). For velocity analyses, fluorescence intensity from regions of interest (50 pixels) on the petiole 13 was analyzed using Fiji/image J (http://fiji.sc/Fiji). Background signal was subtracted from the fluorescence by subtracting the mean intensity of the first 10 frames. Travel velocities of NaFluo were calculated as *V*_Fluo._ = *D*/Δ*t*_ROI2-ROI1_, *D* is the distance between ROI2 and ROI1, Δ*t*_ROI2-ROI1_ is the time difference when fluorescence reaches half-maximum intensity in ROI2 and ROI1.

### Electrophysiology

Electrical signals were recorded on leaf 13 when chemicals were fed by Ricca assay from leaf 8 scalded petiole or 50% of the leaf 8 apical region was crush wounded with forceps. Noninvasive electrophysiology was performed as described by Mousavi et al. (2013). All electrical recordings were performed in a Faraday cage. Two-channel amplifiers (Duo 773, World Precision Instruments, Friedberg, Germany) were used to record surface potential changes with the reference electrode placed in the soil. Five microliter of conducting solution (10 mM KCl in 50% [v/v] glycerol) pipetted onto leaf surface was used to connect the plants to silver electrodes which were chloridized with 0.1 M HCl whenever necessary. Electrical signals acquired at 100 Hz were analyzed using LabScribe4 software (iWorx Systems, Inc., Dover, NH, USA). For the quantification of electrical signals, amplitudes and durations were acquired, where the amplitude is the difference in voltage between baseline and maximum depolarization. Duration is from the time the electrical signals reach half-maximum depolarization voltage to that when they re-reach the half-maximum depolarization voltage during the repolarization phase. Velocities of SWPs were calculated using the following formula: *V*_SWP_ = *D*/Δ*t*_Ed-Ep_, *D* is the distance between distal electrode Ed and proximal electrode Ep, Δ*t*_Ed-Ep_ is the time difference when SWP reaches half-maximum depolarization voltage in Ed and Ep.

### Reverse transcription quantitative PCR and genotyping

Total RNA (1 mg) from leaf 13 was copied into complementary DNA with M-MLV reverse transcriptase, RNase H Minus, Point Mutant (Promega, Dübendorf, Switzerland). The PCR mixture (20 μL) includes 0.5 × SYBR Green I (Invitrogen, Thermo Fisher Scientific), 30 nM ROX reference dye (Thermo Fisher Scientific), 0.5 units GoTaq DNA polymerase (Promega, Dübendorf, Switzerland), 0.2 mM dNTPs, 2.5 mM MgCl_2_, and 0.25 mM of each primer (Microsynth AG, Balgach, Switzerland). Primers for reference gene *UBIQUITIN-CONJUGATING ENZYME 21* (*UBC21*, AT5G25760): forward: 5′-CAGTCTGTGTGTAGAGCTATCATAGCAT-3′; reverse: 5′-AGAAGATTCCCTGAGTCGCAGTT-3′. Primers for *JASMONATE ZIM-DOMAIN 10* (*JAZ10*, AT5G13220): forward: 5′-ATCCCGATTTCTCCGGTCCA-3′; reverse 5′-ACTTTCTCCTTGCGATGGGAAGA-3′. RT-qPCR was performed with an Applied Biosystems QuantStudio 3 Real-Time PCR System (Thermo Fisher Scientific, Reinach, Switzerland). The PCR program was: 2 min initial denaturation step at 95 °C, followed by 40 cycles of 10 s at 95 °C, 30 s at 60 °C, and 30 s at 72 °C. RT-qPCR data were analyzed using the 2^−ΔΔCT^ method. Anion channels examined in this study were genotyped with primers listed in Supplemental Table S2.

### Quantification and statistical analysis

GraphPad Prism 8.0.2 (www.graphpad.com) was used for graph plotting and statistical analyses. Unpaired two-tailed Student's *t*-test or one-way ANOVA followed by Tukey's test were performed for statistical analysis. In graphs with error bars, data are shown as mean ± standard deviation (SD) and *P*-values are indicated. Each experiment was replicated at least twice with similar results. The number (*n*) of replicates or plants used for experiments is indicated for each figure.

## Supplementary Material

kiad584_Supplementary_Data

## Data Availability

All data generated during this study are presented in this paper and in the supplemental files.

## Supplemental data

The following materials are available in the online version of this article.

**Supplemental Figure S1.** Scalding induces no electrical signals and only weak *JAZ10* expression in distal leaf 13.

**Supplemental Figure S2.** Long-distance transportation of Na-Fluorescein fed into unscalded (healthy) and scalded petioles.

**Supplemental Figure S3.** Effect of time after scalded petiole cutting on FLE-induced electrical signaling.

**Supplemental Figure S4.** Proton NMR identifies glutamate in a depolarization-inducing fraction.

**Supplemental Figure S5.** The activity of amino acids in inducing electrical signals.

**Supplemental Figure S6.** *JAZ10* transcript induction by glutathiones.

**Supplemental Figure S7.** Wound-induced SWPs in *pad2-1*.

**Supplemental Table S1.** Wound-induced SWPs in *clc* and *almt* mutants.

**Supplemental Table S2.** Accession numbers and primers for genotyping.

**Supplemental Movie S1.** Long-distance transportation of Na-Fluorescein fed into severed unscalded petioles.

**Supplemental Movie S2.** Long-distance transportation of Na-Fluorescein fed into severed scalded petioles.
