# Supplementary material for: Chloride, glutathiones, and insect-derived elicitors introduced into the xylem trigger electrical signaling
Source: Plant Physiol. 2023 Oct 31;194(2):1091–103. doi: 10.1093/plphys/kiad584 (PMC10828190; doi:10.1093/plphys/kiad584)
Supplement: kiad584_Supplementary_Data [file kiad584_supplementary_data.zip › PP2023RA01263DR1_Supplemental_Figures_1_7.pdf]

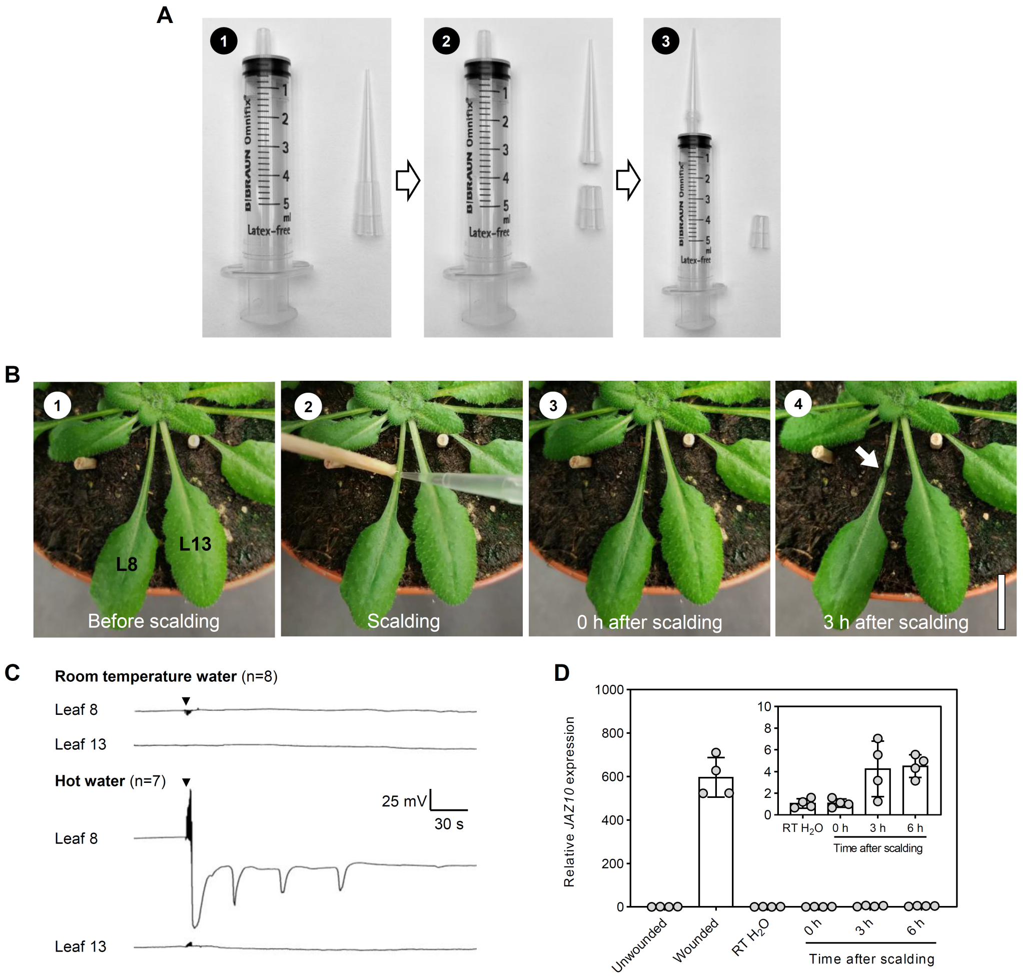

Supplemental Figure S1. Scalding induces no electrical signals and only weak *JAZ10* expression in distal leaf 13.

A. The procedure of syringe + pipette tip assembly. A 5 mL syringe and a 200 µL pipette tip were used.

B. Procedure for petiole 8 scalding. A toothpick was used to drain water into the soil. The pipette tip on the right was connected to a syringe to apply hot water. The white arrow indicates the shrinking petiole. Scale bar = 1 cm; all panels photographed at same magnification.

C. Typical electrical signals recorded on leaf 8 and on leaf 13. Numbers in parentheses represent the number of total recordings. The electrode on petiole 8 was placed between the position of hot water application and the rosette center.

D. *JAZ10* expression analyses in distal leaf 13 after scalding of petiole 8 (n=4, means ± SD). Leaf 8 was crush-wounded and analysed 1 h after wounding. Room temperature water (RT H_2O) applied as the negative control. Inset, magnification of RT H_2O and scalding induced *JAZ10* expression.

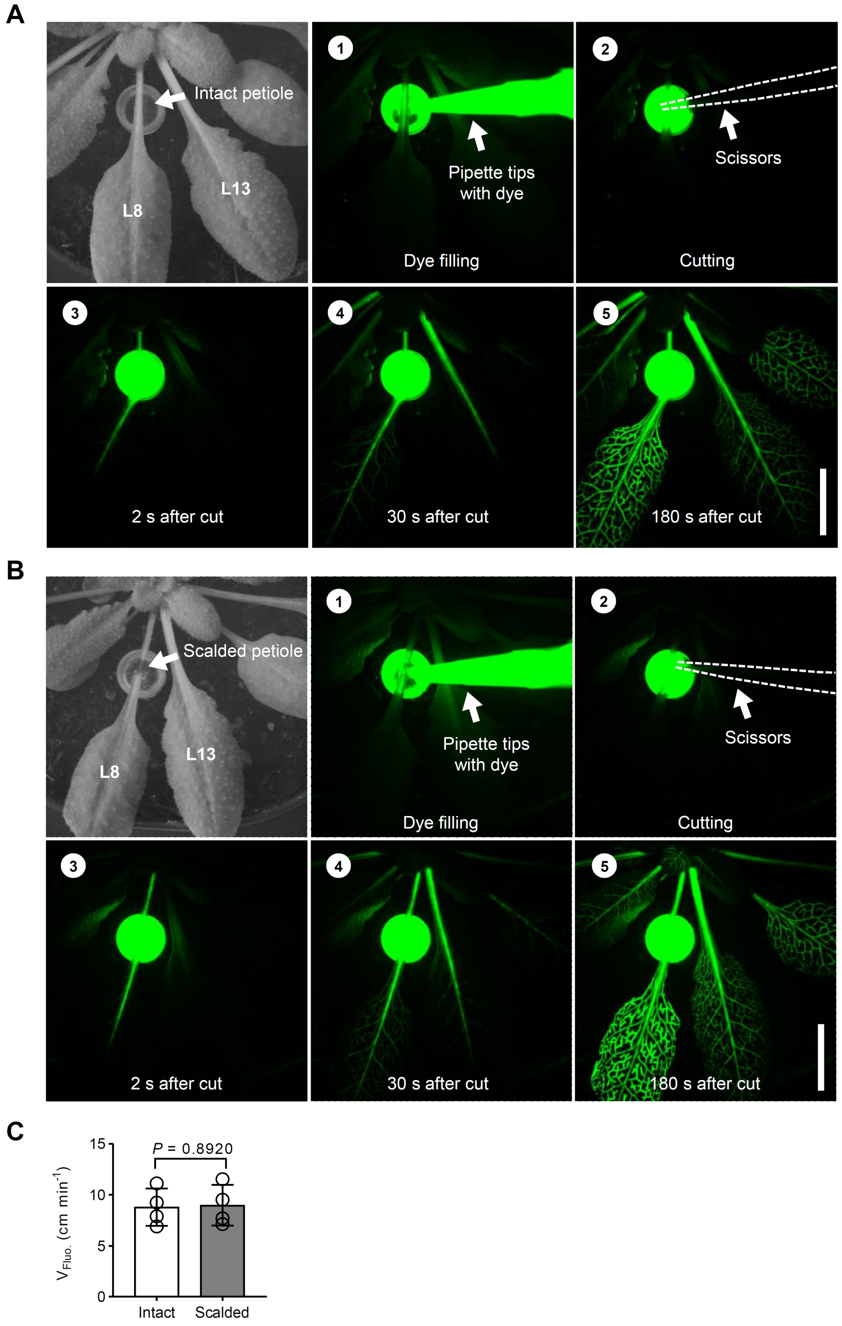

Supplemental Figure S2. Long-distance transportation of Na-Fluorescein fed into unscalded (healthy) and scalded petioles.

A. Na-Fluorescein (NaFluo) feeding through an unscalded petiole. Scale bar = 1 cm.

B. NaFluo feeding through the scalded petiole. Plants were used 3 h after scalding. Scale bar = 1 cm. All images in panels A and B were obtained at the same magnifications.

C. Velocity of NaFluo propagation in distal leaf 13 (n=4, means ± SD, unpaired two-tailed Student's *t*-test). The significance threshold is $P < 0.05$.

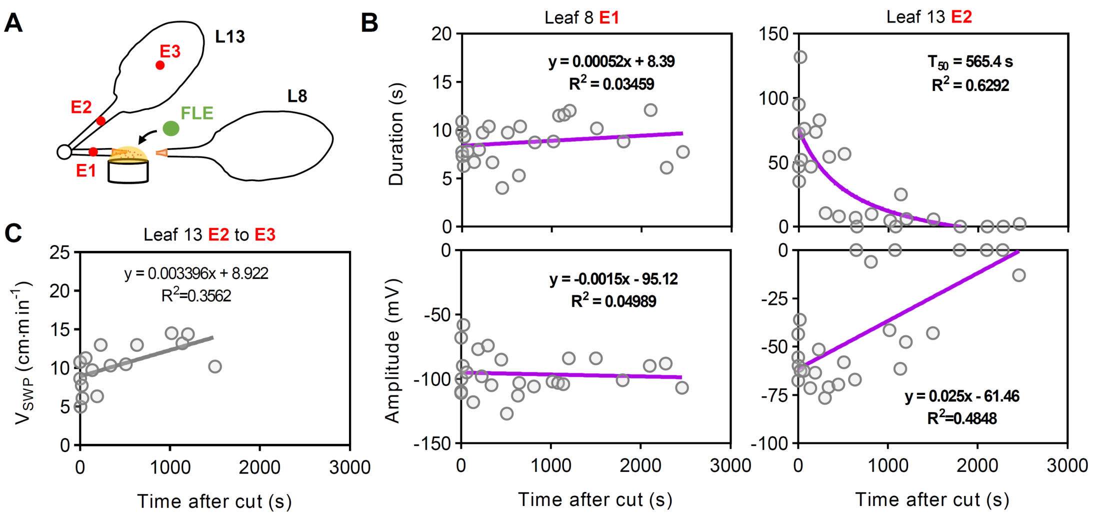

Supplemental Figure S3. Effect of time after scalded petiole cutting on FLE-induced electrical signaling.

A. Experiment design of fresh leaf extract (FLE) application into petiole of leaf 8 (L8). The scalded petiole was immersed in 40 μL 50 mM MES-Tris, pH 6.0 and cut with scissors, then 10 μL 1×FLE was added, the final concentration of FLE was 0.2×. The positions of electrodes are shown as red dots. L13=leaf 13.

B. Electrical signals recorded on leaf 8 (left) and on leaf 13 petiole (right, n=26).

C. Velocity of slow wave potential (SWP) propagation from E2 to E3 in distal leaf 13 (n=17).

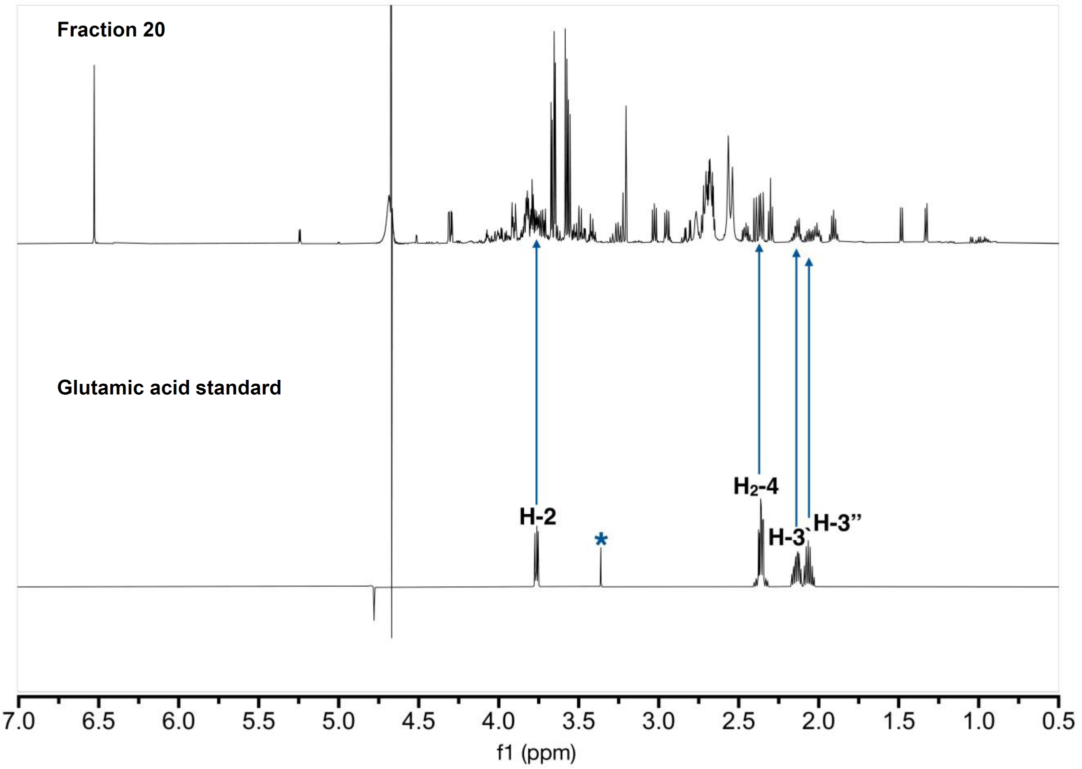

Supplemental Figure S4. Proton NMR identifies glutamate in a depolarization-inducing fraction.

^{1}H NMR spectrum of the active fraction 20 (upper part) obtained after size exclusion chromatography stacked with ^{1}H NMR spectra of the glutamic acid standard (lower part). The characteristic signals (chemical shift and multiplicity, blue arrows) of glutamic acid were observed in the mixture of compounds of fraction 20. The signal at 3.3 ppm in the standard corresponds to residual methanol (blue asterisk).

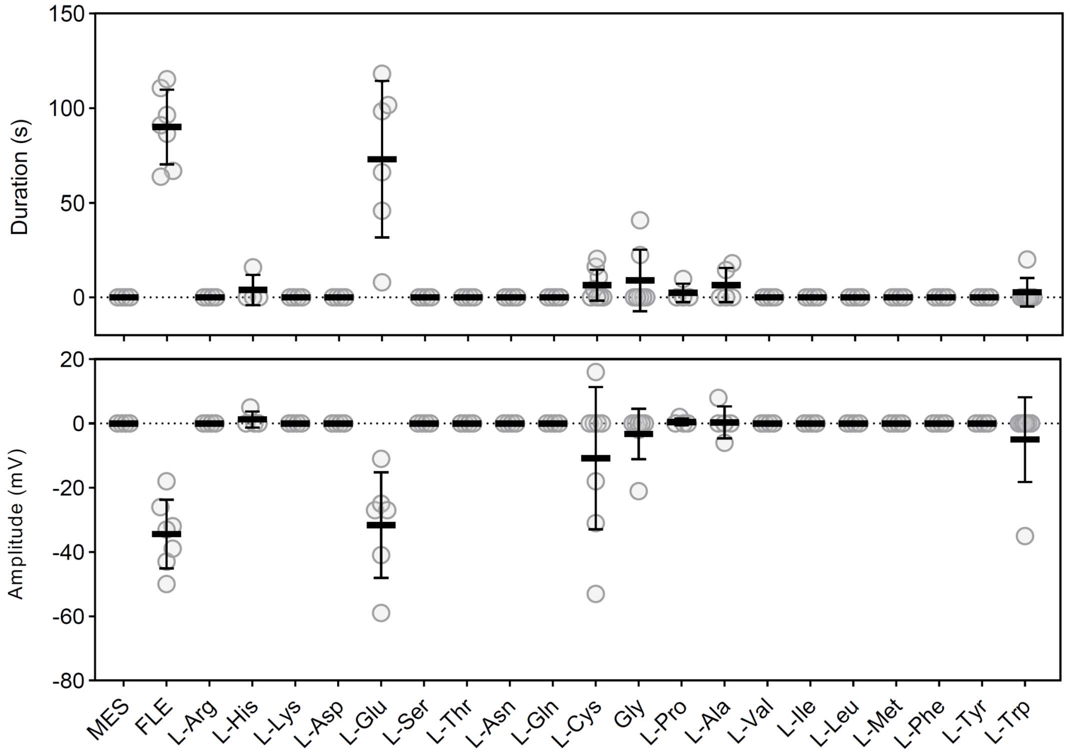

Supplemental Figure S5. The activity of amino acids in inducing electrical signals. Amino acids were dissolved in 50 mM MES, pH 6.0 with Tris at a concentration of 5 mM, except L-Tyr (2.5 mM) (n=4-7, means ± SD). Amino acids were applied from leaf 8 and slow wave potentials (SWPs) were recorded on leaf 13 with the surface electrodes.

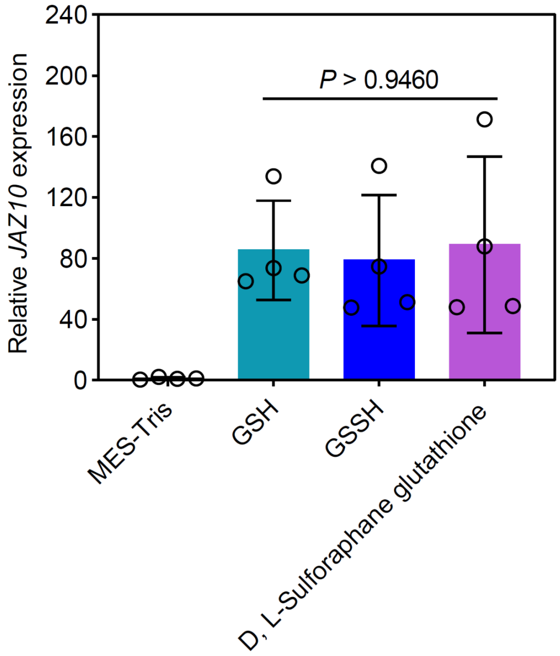

Supplemental Figure S6. *JAZ10* transcript induction by glutathiones.

5 mM GSH, 5 mM GSSH, and 20 mM D, L-Sulforaphane glutathione-induced *JAZ10* expression in wild-type plants (n = 4, means ± SD; one way ANOVA followed by Tukey's test for multiple comparisons). Significance threshold $P < 0.05$. Chemicals were applied through leaf 8 with the Ricca assay and leaf 13 was sampled 1 hour after treatment for analyses.

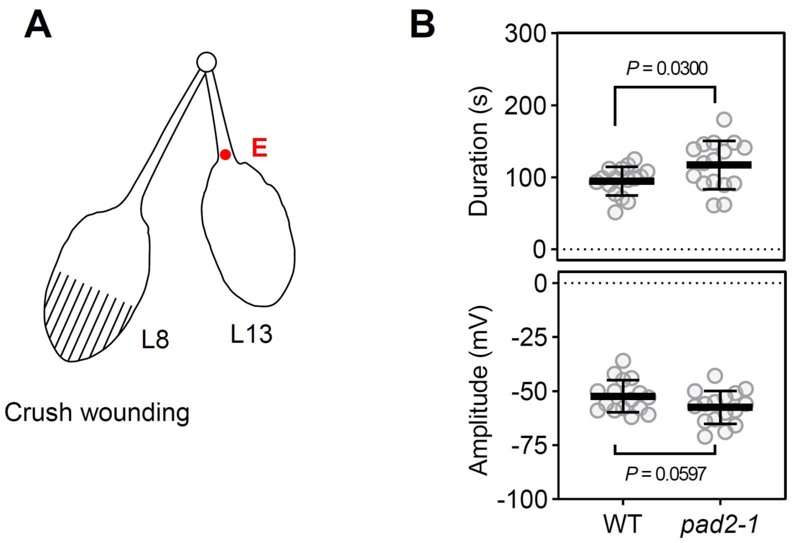

Supplemental Figure S7. Wound-induced slow wave potentials in *pad2-1*.

A. Experimental design for crush wounding leaf 8 and electrical signal detection in leaf 13 (red dot, surface electrode).

B. Crush wounding-induced slow wave potentials (n = 16, means ± SD; unpaired two-tailed Student's *t*-test). Significance threshold $P < 0.05$.

Supplemental Table S1. Slow wave potentials in *clc* and *almt* mutants.

Name	Amplitude (mV)	Duration (s)	n =
WT	-49 ± 10	72 ± 30	11
clc-b	-50 ± 11 (0.7140)	61 ± 19 (0.3361)	11
clc-g	-58 ± 15 (0.1255)	65 ± 20 (0.5637)	10
WT	-49 ± 11	139 ± 21	9
almt1	-42 ± 10 (0.1850)	142 ± 29 (0.8687)	8
WT	-59 ± 6	110 ± 22	9
almt3	-46 ± 7 (0.0008)	116 ± 32 (0.6804)	9
almt4	-60 ± 8 (0.6828)	107 ± 55 (0.8862)	11
WT	-65 ± 5	96 ± 41	9
almt6	-65 ± 6 (0.9410)	69 ± 47 (0.1853)	11
WT	-60 ± 7	102 ± 39	11
almt13	-64 ± 7 (0.3274)	95 ± 17 (0.6271)	11

Note: Data are shown as means ± SD, unpaired two-tailed Student's *t*-test was used for the statistical analysis of mutant VS WT, *P* values are shown in the parentheses.

Supplemental Table S2. Accession numbers and primers for genotyping.

Name	Gene ID	Mutant ID	Primers for genotyping
clc-b	AT3G27170	SALK_027349	LP: TCAACCCGTGGAGTTCTGTAG<br>RP: GGAATTCTTGGGAGCCTGTAC
clc-g	AT5G33280	SALK_087699	LP: AATTTCCGAGCTCTATCGCTC<br>RP: CGTCTACCTCCTTTTTCCATTTC
almt1	AT1G08430	SALK_009629	LP: GAAATTATTTGGGGAAGCTGC<br>RP: TCTTTACCCATGGGAAAAACC
almt3	AT1G18420	SALK_013699	LP: AACAGTGTTCATGGAGATGCC<br>RP: CTACGAGATTCACCAAGCAGC
almt4	AT1G25480	SALK_119455	LP: TAATGGATCATCCGAAGCTTG<br>RP: TCTCTCGAAAAGCTTGCTCAG
almt6	AT2G17470	SALK_205696	LP: TTCTGCAAACCTAATTTTGCG<br>RP: AAGTGTGTCCTTCATTGGTGG
almt13	AT5G46600	SAIL_517F03	LP: TAATAAAGCGGAATTGGAACG<br>RP: GGCGGATTTAGAATCCAAAAC
