# Supplementary material for: Chloride, glutathiones, and insect-derived elicitors introduced into the xylem trigger electrical signaling
Source: Plant Physiol. 2023 Oct 31;194(2):1091–103. doi: 10.1093/plphys/kiad584 (PMC10828190; doi:10.1093/plphys/kiad584)
Supplement: kiad584_Supplementary_Data [file kiad584_supplementary_data.zip › PP2023RA01263DR1_Supplemental_movie_legends.pdf]

Supplemental Movie S1. Long-distance transport of Na-Fluorescein fed into severed unscalded petioles.

A healthy petiole was cut with scissors into Na-Fluorescein (1 mg mL^{-1}) in water.

Supplemental Movie S2. Long-distance transport of Na-Fluorescein fed into severed scalded petioles.

A scalded petiole was severed across the scald site with scissors in Na-Fluorescein (1 mg mL^{-1}) in water. The petiole was cut 3 hours after scalding.
